# Regulation of serine palmitoyl-transferase and Rac1–Nox2 signaling in diabetic retinopathy

**DOI:** 10.1038/s41598-022-20243-2

**Published:** 2022-10-06

**Authors:** Kumari Alka, Ghulam Mohammad, Renu A. Kowluru

**Affiliations:** Ophthalmology, Visual and Anatomical Sciences, Kresge Eye Institute, Wayne State University, Detroit, MI 48201 USA

**Keywords:** Cell biology, Medical research

## Abstract

Hyperlipidemia is considered as one of the major systemic factors associated with the development of diabetic retinopathy, and animal models have documented that its presence in a hyperglycemic environment exacerbates cytosolic ROS production (via activation of the Rac1–Nox2 axis) and mitochondrial damage. Hyperglycemia also accelerates *Rac1* transcription via dynamic DNA methylation–hydroxymethylation of its promoter. In diabetes, ceramide metabolism in the retina is impaired and its accumulation is increased. Our aim was to investigate the effect of inhibition of the rate limiting enzyme of the de novo ceramide biosynthesis, serine palmitoyl-transferase (SPT), on *Rac1* activation in diabetic retinopathy. Using human retinal endothelial cells, transfected with *SPT*-siRNA, and incubated in 20 mM d-glucose in the presence or absence of 50 µM palmitate (glucolipotoxic and glucotoxic, respectively), activities of Rac1 and Nox2, and ROS levels were quantified. For *Rac1* transcriptional activation, 5 hydroxymethyl cytosine (5hmC) levels at its promoter were quantified. Key parameters were confirmed in retinal microvessels from streptozotocin-induced diabetic mice on a normal diet (type 1 diabetic model) or on a high-fat diet (45% kcal, type 2 diabetic model), injected intravitreally with *SPT*-siRNA. Compared to normal glucose, cells in high glucose, with or without palmitic acid, had increased Rac1–Nox2–ROS signaling, *Rac1* transcripts and 5hmC levels at its promoter. Inhibition of SPT by *SPT*-siRNA or myriocin prevented glucotoxic- and glucolipotoxic-induced increase in Rac1–Nox2–ROS signaling and 5hmC at the *Rac1* promoter. Similarly, in both type 1 and type 2 diabetic mouse models, *SPT*-siRNA attenuated the increase in the Rac1–Nox2–ROS axis and 5hmC at the *Rac1* promoter. Thus, inhibition of the rate limiting enzyme of ceramide de novo biosynthesis, SPT, regulates activation of DNA methylation–hydroxymethylation machinery and prevents increased *Rac1* transcription. This ameliorates the activation of Rac1–Nox2 signaling and protects the mitochondria from damaging cytosolic ROS, which prevents accelerated capillary cell loss. These results further raise the importance of regulating lipid levels in diabetic patients with dyslipidemia.

## Introduction

Diabetes is now considered an epidemic of the twentieth century, and claims over four million lives every year. Over 90% of patients have type 2 diabetes, which is closely linked to obesity and a sedentary lifestyle. Diabetic patients suffer from many vascular complications, and retinopathy is one of the major microvascular complications of diabetes feared the most by a diabetic patient^1,2^. Clinical and experimental models have documented hyperglycemia as the instigator of diabetic retinopathy^3–5^. However, dyslipidemia is also now considered one of the major systemic factors playing a critical role in diabetic retinopathy, and animal models have shown that hyperglycemia, in a hyperlipidemic environment accelerates capillary cell apoptosis and the development of diabetic retinopathy^6–8^.

In the pathogenesis of diabetic retinopathy, an increase in cytosolic reactive oxygen species (ROS), generated by the activation of a small G-protein Ras-related C3 botulinum toxin-substrate (Rac1)-NADPH oxidase 2 (Nox2) signaling is an early event, that damages mitochondria and accelerates capillary cell loss^7,8^. Our previous work has shown that the presence of hyperlipidemia in a hyperglycemic milieu (type 2 diabetic model) further exacerbates the activation of Rac1–Nox2–ROS signaling, and accelerates the development of diabetic retinopathy by accelerating and exacerbating mitochondrial damage-apoptosis^7–10^. Furthermore, the *Rac1* promoter also undergoes dynamic DNA methylation; despite activation of DNA methylating enzymes and increased binding of DNA methyltransferase 1 (Dnmt1), concomitant activation of ten-eleven translocation methylcytosine dioxygenases (Tets) quickly converts 5 methyl cytosine into 5-hydroxymethyl cytosine (5hmC), and increased 5hmC at *Rac1* promoter facilitates binding of the transcription factors and activates *Rac1* transcription^7,8,11,12^.

Individuals with dyslipidemia or obesity also have higher ectopic accumulation of lipid metabolite ceramide in non-adipose tissues including heart and blood vessels, and ceramides can modulate signaling and metabolic pathways, further increasing triglyceride production and tissue damage^13,14^. In diabetes, ceramide levels are upregulated in the retina^15,16^, and the addition of ceramide in a hyperglycemic medium further accentuates hyperglycemia-induced activation of Rac1–Nox2–ROS signaling and mitochondrial damage^17^. Ceramide is considered as the precursor of most complex sphingolipids, and its biosynthesis is a multistep process. The first step in its de novo biosynthesis is the condensation of palmitoyl-CoA and serine to produce 3-ketosphinganine in the endoplasmic reticulum, which is catalyzed by the rate-limiting enzyme serine palmitoyl transferase (SPT). With the help of vesicular trafficking or ceramide transfer proteins, ceramide is transported to the Golgi bodies^18–20^. In diabetes, ceramide metabolism is dysregulated in the retina and formation of proinflammatory and proapoptotic short-chain ceramide is elevated^16^.

This study aims to investigate the effect of inhibition of SPT on Rac1 activation in diabetic retinopathy. Using human retinal endothelial cells (HRECs), we investigated the effect of regulation of SPT on Rac1 functional and transcriptional activation. The results were confirmed by intravitreally administrating *SPT*-siRNA in type 1 and type 2 diabetic models that develop histopathology characteristic of diabetic retinopathy^7,21^.

## Results

### Retinal endothelial cells

#### Serine palmitoyl-transferase

**Figure 1:**
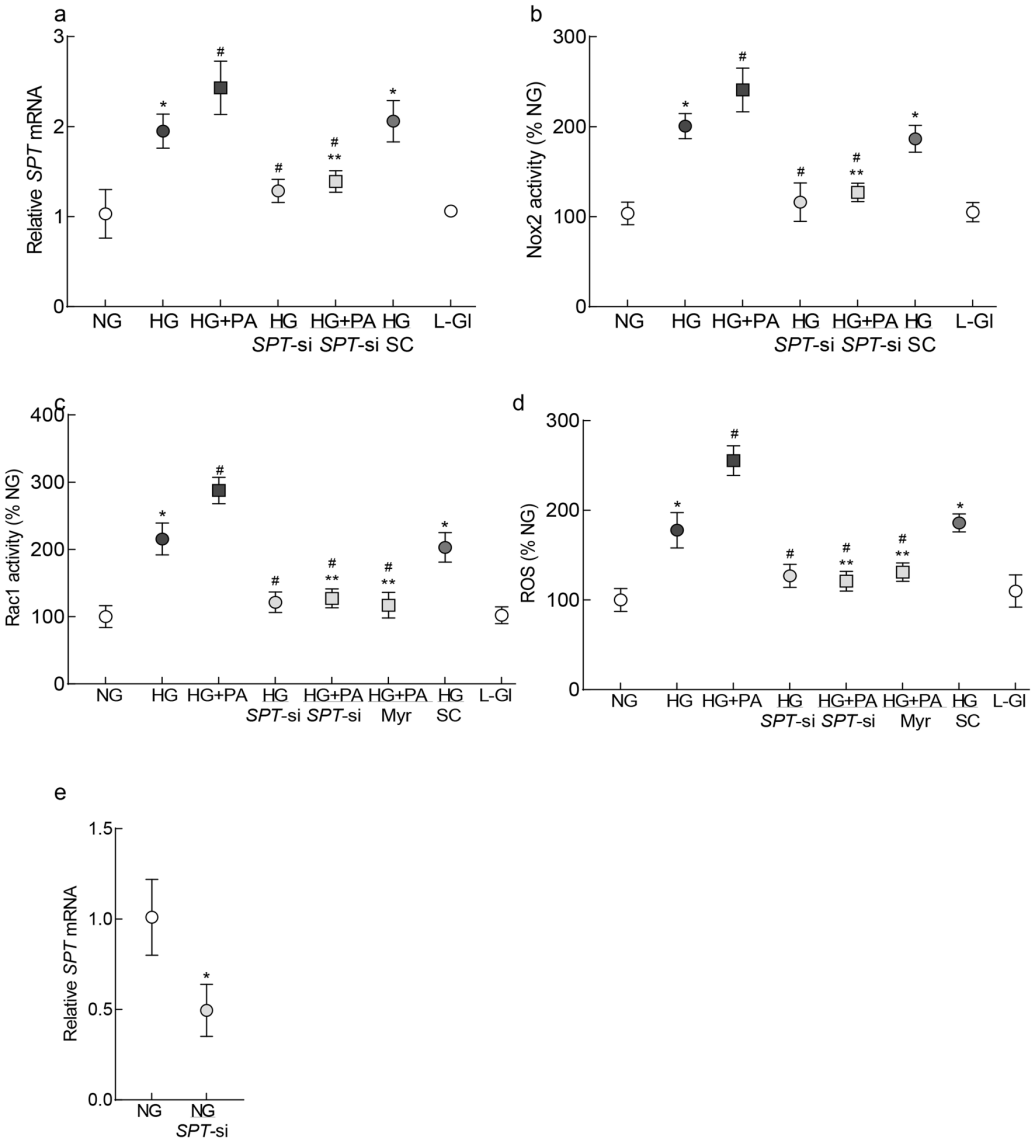
Inhibition of SPT and Rac1–Nox2–ROS signaling in HRECs. (**a**) *SPT* mRNA was quantified by qRT-PCR using β-actin as a house keeping gene. (**b**) Nox2 by using lucigenin as an electron acceptor and NADPH as a substrate and (**c**) Rac1 activity was determined by G-LISA colorimetric assay kit. (**d**) ROS levels were quantified by a fluorescence method using DCHFDA. (**e**) Transfection efficiency of *SPT*-siRNA was determined by quantifying the relative mRNA of *SPT*. Each measurement was made in duplicate, and the values are represented as mean ± SD from 3–4 cell preparations. NG and HG = 5 mM or 20 mM d-glucose; HG + PA = 20 mM d-glucose + palmitic acid; HG/*SPT*-si and HG + PA/*SPT*-si = *SPT*-siRNA transfected cells in 20 mM d-glucose or 20 mM d-glucose + palmitic acid; HG + PA/Myr = 20 mM d-glucose + palmitic acid + Myriocin; HG/SC = *SPT*-scrambled control RNA transfected cells in 20 mM d-glucose; L-Gl = 20 mM l-glucose. *p < 0.05 vs NG, ^#^p < 0.05 vs HG and ^#^**p < 0.05 vs HG + PA.

Compared to normal glucose (5 mM d-glucose, NG group), HRECs incubated in high glucose (20 mM d-glucose, HG group, glucotoxic) had about two-fold increase in *SPT* mRNA levels. *SPT* mRNA was further increased in a glucolipotoxic environment when high glucose medium was supplemented with 50 µM palmitic acid (HG + PA group; p < 0.05 vs HG). Regulation of *SPT* by its siRNA (*SPT*-si) ameliorated glucose or glucose + PA-induced increase in *SPT* mRNA, and the values in these two groups were not different from those obtained from cells in normal glucose (p < 0.05 vs NG). As an osmotic/metabolic control, cells incubated in 20 mM l-glucose (L-Gl group), instead of 20 mM d-glucose, had values similar to those obtained from cells in normal glucose (Fig. 1a).

#### Rac1–Nox2–ROS signaling

Compared to the NG group, as expected^17^, exposure of HRECs to high glucose activated Rac1–Nox2 signaling, and elevated ROS levels. Activation of Rac1–Nox2–ROS was further exacerbated in a glucolipotoxic environment (HG + PA group; Fig. 1b–d). Transfection of cells with *SPT-* siRNA or supplementation of medium with 5 μM Myriocin (Myr) ameliorated glucolipotoxicity-induced exacerbated Rac1 and Nox2 activation, and increase in ROS, the values obtained from *SPT*-siRNA transfected cells and untransfected cells in glucolipotoxic medium (HG + PA/*SPT*-si and HG + PA groups respectively) were significantly different from each other (p < 0.05). However, scrambled control RNA transfected cells or untransfected cells, incubated with high glucose, had similar values. Cells incubated in 20 mM l-glucose (L-Gl group), instead of 20 mM d-glucose, had values similar to those obtained from cells in 5 mM d-glucose, ruling out the effect of osmolarity on any of these parameters. Figure 1e shows 50% reduction in *SPT* gene in HRECs by *SPT*-siRNA.

**Figure 2:**
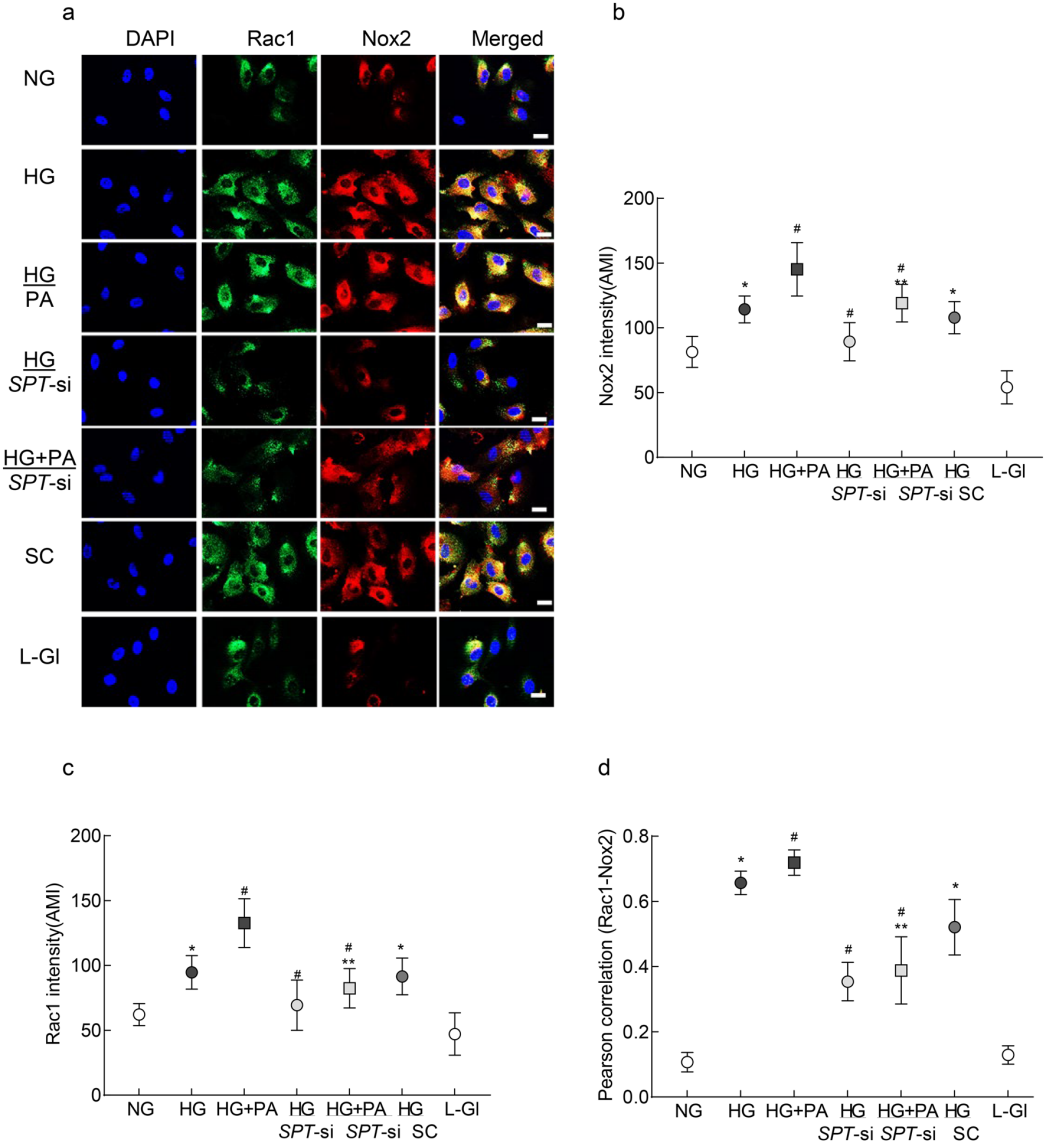
Rac1–Nox2 colocalization and inhibition of SPT. (**a**) Rac1 and Nox2 were determined by immunofluorescence using DyLight green (green)-conjugated and Texas Red (red)-conjugated secondary antibodies, respectively. The line marker represents 20µm. ‘Arithmetic mean intensity’ (AMI) of (**b**) Nox2 and (**c**) Rac1 was quantified by Zeiss software, from 5 to 8 images/group. (**d**) Zeiss software module was used to calculate Pearson’s correlation coefficient between Rac1 and Nox2. The values in the graph are mean ± SD, obtained from 3–4 different cell preparations with multiple cells analyzed in each preparation. *p < 0.05 vs NG, ^#^p < 0.05 vs HG and ^#^**p < 0.05 vs HG + PA.

Consistent with Rac1–Nox2 axis, exposure of cells to high glucose or high glucose + palmitic acid increased immunofluorescence for both Rac1 and Nox2 (Fig. 2a–c), and Pearson’s correlation coefficient of Rac1–Nox2 in these two groups was significantly higher compared to cells in NG group (Fig. 2d). *SPT*-siRNA attenuated glucotoxicity- or glucolipotoxicity-induced Rac1–Nox2 co-localization; and the values in HG/*SPT*-si and HG + PA/*SPT*-si groups were not different from each other (p > 0.05; Fig. 2).

#### Mitochondrial damage-cell death

**Figure 3:**
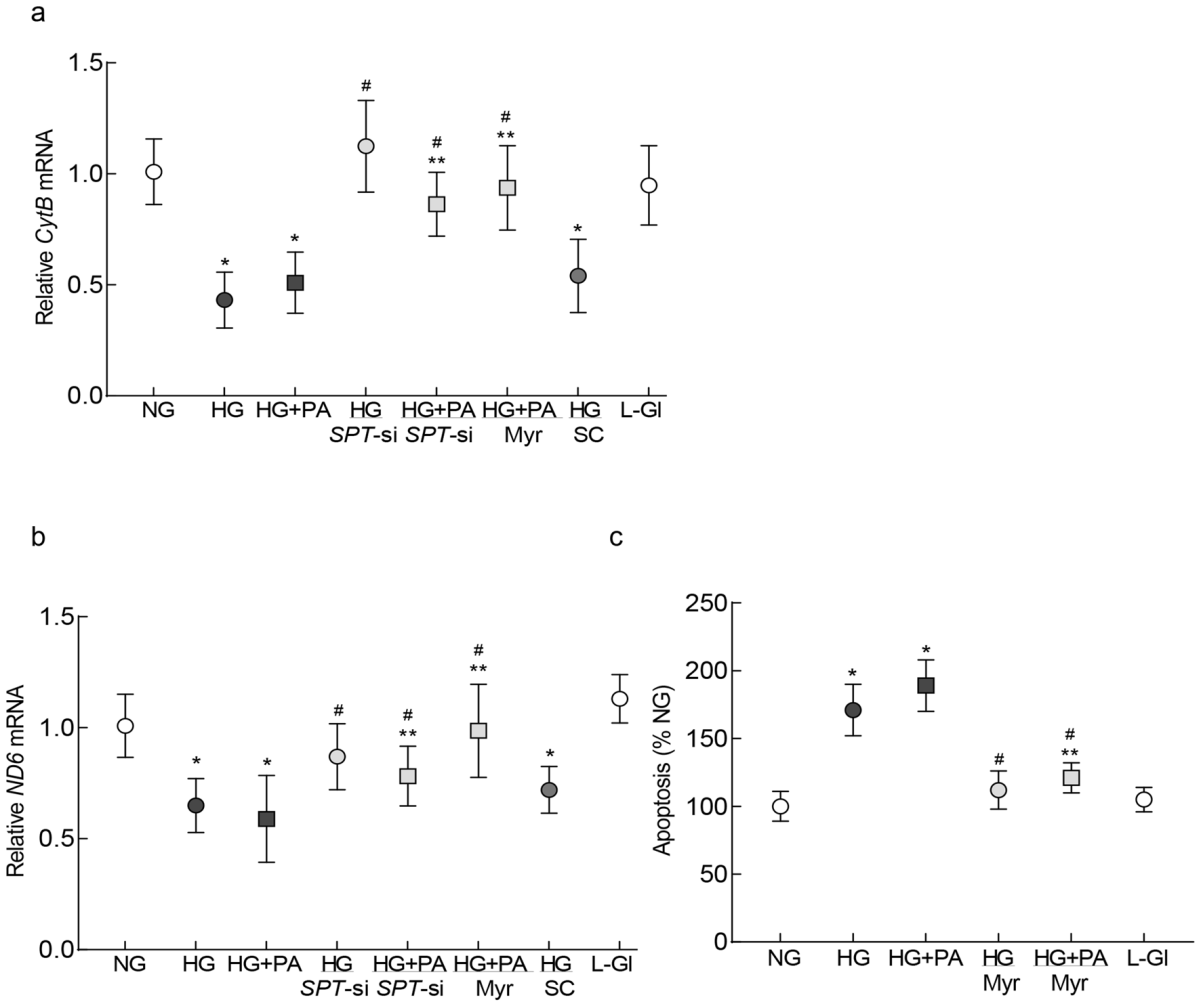
Regulation of SPT and mitochondrial damage-cell apoptosis. Damage of mtDNA was determined by quantifying relative mRNA levels of mtDNA-encoded (**a**) *CytB* and (**b**) *ND6* by qRT-PCR, β-actin was employed as a housekeeping gene. (**c**) Cell death was determined using a Cell Death Detection ELISA PLUS kit. Each measurement was made in duplicate, and the values are represented as mean ± SD from 3 or more cell preparations. NG and HG = 5 mM or 20 mM d-glucose; HG + PA = 20 mM d-glucose + palmitic acid; HG/*SPT*-si and HG + PA/*SPT*-si = *SPT*-siRNA transfected cells in 20 mM d-glucose or 20 mM d-glucose + palmitic acid; HG + PA/Myr = 20 mM d-glucose + palmitic acid + Myriocin; HG/SC = *SPT*-scrambled control RNA transfected cells in 20 mM d-glucose; L-Gl = 20 mM l-glucose. *p < 0.05 vs NG, ^#^p < 0.05 vs HG and ^#^**p < 0.05 vs HG + PA.

In the development of diabetic retinopathy, activation of Rac1–Nox2–ROS signaling precedes mitochondrial damage-capillary cell apoptosis^4,7^; the effect of regulation of inhibition of SPT on mitochondrial damage and cell death was evaluated. Consistent with our previous results^7,10^, gene transcripts of mtDNA-encoded *cytochrome B* (*CytB*) of complex III and *ND6* of complex I were significantly reduced and cell death was accelerated in cells incubated in high glucose, compared to normal glucose (Fig. 3a–c). Although, compared to the HG group, the addition of palmitic acid in high glucose medium further reduced gene transcripts of *CytB* and *ND6,* and increased cell death, the values in HG + PA and HG groups were not significantly different from each other (p > 0.05 vs HG). Inhibition of SPT by either *SPT*-siRNA or Myr ameliorated decrease in *ND6* and *CytB* gene transcripts and increase in cell death. The values obtained from cells in 20 mM l-glucose were similar to those obtained from cells in normal glucose.

#### Inhibition of SPT and *Rac1* transcription

**Figure 4:**
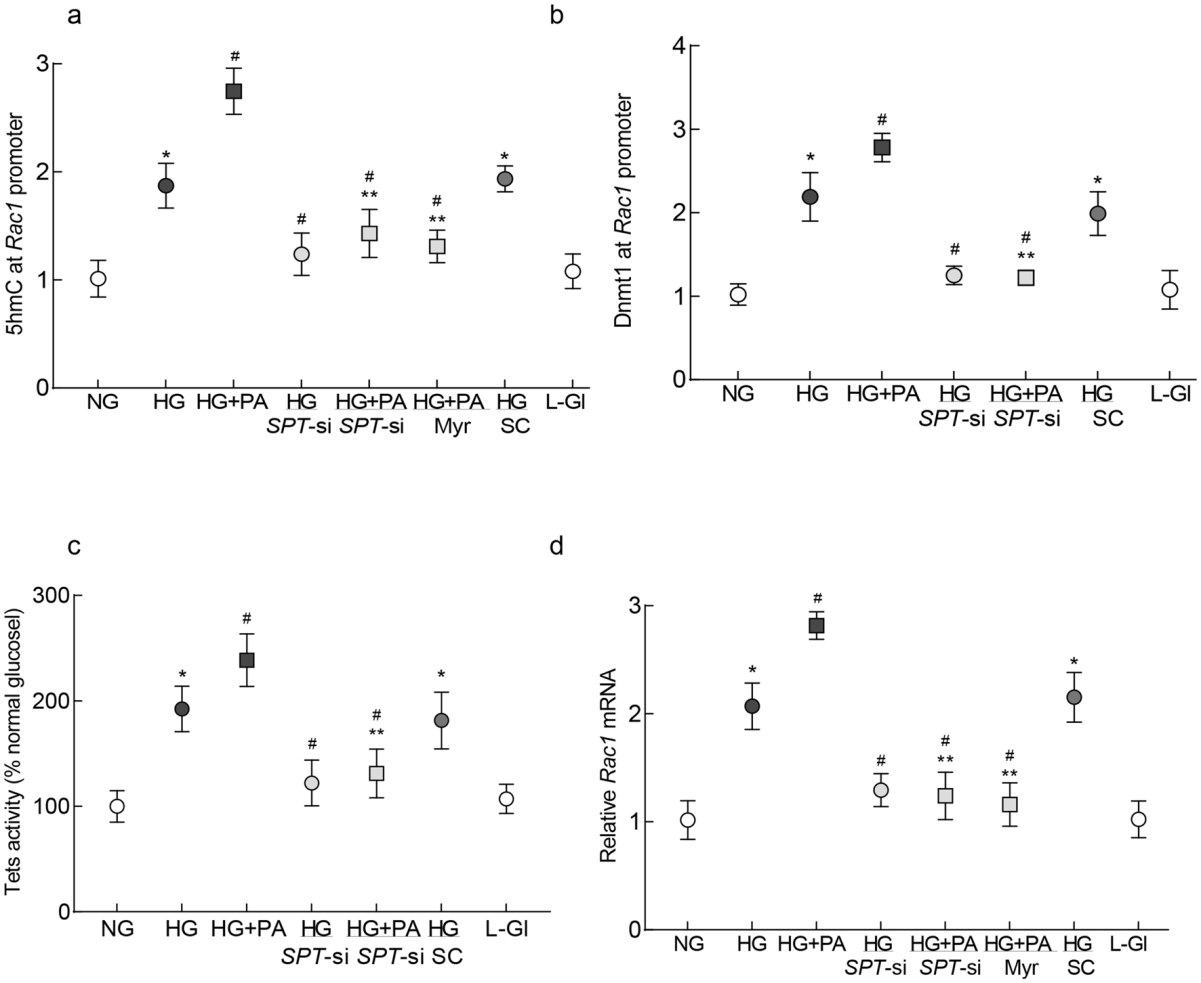
Regulation of SPT and transcriptional activation of *Rac1* in HRECs. (**a**) 5hmC levels at *Rac1* promoter were quantified using hydroxyl-methylated DNA immunoprecipitation technique, and (**b**) binding of Dnmt1 at *Rac1* promoter by ChIP assay using IgG as a negative antibody control. (**c**) Activity of Tets was measured by TET Activity/Inhibition Assay Kit, and (**d**) relative *Rac1* mRNA by qRT-PCR using β-actin as a housekeeping gene. Values are represented as mean ± SD from 3–4 cell preparations, with each measurement made in duplicate. NG and HG = 5 mM or 20 mM d-glucose; HG + PA = 20 mM d-glucose + palmitic acid; HG/*SPT*-si and HG + PA/*SPT*-si = *SPT*-siRNA transfected cells in 20 mM d-glucose or 20 mM d-glucose + palmitic acid; HG + PA/Myr = 20 mM d-glucose + palmitic acid + Myriocin; HG/SC = *SPT*-scrambled control RNA transfected cells in 20 mM d-glucose; L-Gl = 20 mM l-glucose. *p < 0.05 vs NG, ^#^p < 0.05 vs HG and ^#^**p < 0.05 vs HG + PA.

Our previous work has shown that *Rac1* transcription is mediated by DNA methylation–hydroxymethylation of its promoter, and the lipotoxic environment exacerbates glucose-induced increase in 5hydroxymethyl cytosine (5hmC) levels at its promoter^7^. To investigate whether SPT inhibition has any role in altering the DNA methylation status of the *Rac1* promoter and its gene transcription, the effect of *SPT*-siRNA on DNA hydroxymethylation at the *Rac1* promoter was evaluated. As shown in Fig. 4a, compared to untransfected cells, *SPT*-siRNA attenuated glucolipotoxicity-induced increase in 5hmC at *Rac1* promoter. This was accompanied by an increase in the binding of the Dnmt1 at the promoter and increase in the activity of Tets and *Rac1* gene transcripts (Fig. 4b–d), suggesting a possible role of ceramide in DNA methylation–hydroxymethylation of the *Rac1* promoter. Dnmt1 binding at the *Rac1* promoter using IgG antibody control was > 0.1% of the values obtained from Dnmt1 antibody. The values from *SPT*-siRNA transfected cells in glucotoxic or glucolipotoxic medium were not different from each other (p > 0.05), but were significantly different from their respective untransfected cells (p < 0.05). Scrambled control RNA transfected cells, incubated with high glucose, had similar values as those obtained from untransfected cells in high glucose. Cells in 20 mM l-glucose, instead of 20 mM d-glucose, had similar DNA methylation–hydroxymethylation status of *Rac1* promoter similar to that observed in the cells incubated in 5 mM d-glucose.

### Diabetic mice

#### Establishment of type 1 and type 2 diabetic animal models

**Figure 5:**
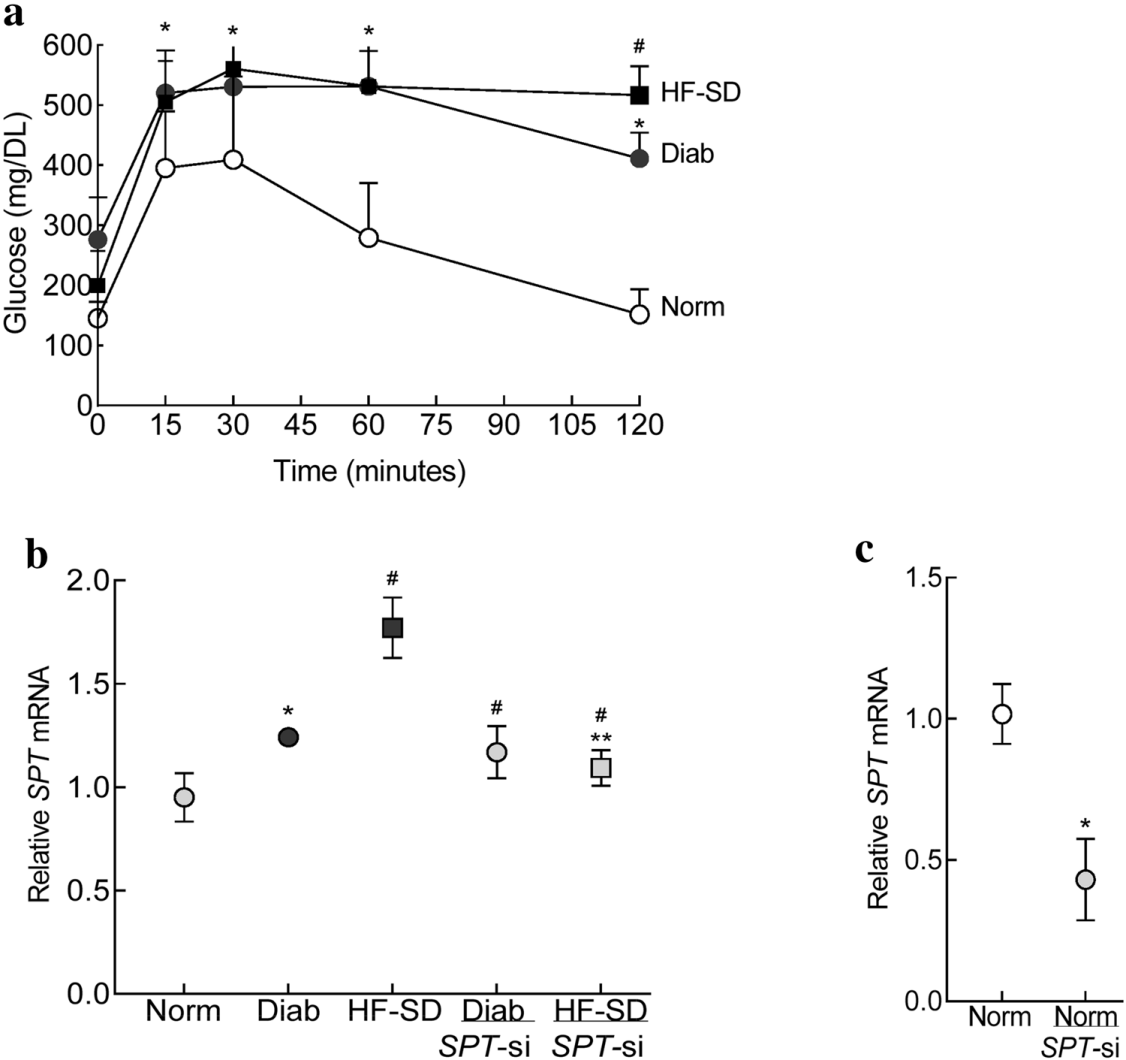
Effect of diabetes on SPT in mice retinal microvessels. (**a**) Glucose utilization was performed in overnight fasted mice. (**b**) Gene transcripts of *SPT* and (**c**) transfection efficiency of *SPT*-siRNA was determined by quantifying relative *SPT* mRNA by qRT-PCR using 18S as a housekeeping gene. Each measurement was made in duplicate 5–7 mice/group, and the values are represented as means ± SD. Norm = normal mice on regular chow; Diab = diabetic mice on regular chow; HF-SD = diabetic mice on high fat chow; Diab*/SPT*-si or HF-SD/*SPT*-si = *SPT*-siRNA administered diabetic mice on regular chow or high fat chow, respectively. *p < 0.05 vs Norm, ^#^p < 0.05 vs Diab and ^#^**p < 0.05 vs HF-SD.

Glucose tolerance test, performed 2–3 days prior to the termination of the experiment, showed impaired glucose utilization even two hours after its infusion, with high glucose values in both streptozotocin-induced type I diabetic mice (Diab group) and high fat-low dose streptozotocin, type 2 diabetic mice (HF-SD group). However, compared to Diab group, the HF-SD group showed significantly higher severity of impairment in glucose utilization (p < 0.05; Fig. 5a). Compared to the Diab group (22.8 ± 3 g) or Norm group (26.9 ± 2 g), the body weights of mice, measured at the time of glucose tolerance test, were significantly higher in HF-SD group (48.5 ± 6 g, p < 0.05).

#### Serine palmitoyl-transferase

Compared to the Norm group, *SPT* gene transcripts were significantly elevated in mice in the Diab and HF-SD groups (p < 0.05 vs Norm), however, increase in *SPT* in the HF-SD group was higher compared to Diab group (p < 0.05 vs Diab; Fig. 5b). Administration of *SPT*-siRNA soon after induction of diabetes prevented an increase in *SPT* in diabetic mice on normal diet or high fat diet (Diab/*SPT*-si and HF-SD/*SPT*-si groups, respectively), and the values in these two groups were not different from those obtained from mice in Norm group (p > 0.05 vs Norm). Figure 5c shows transfection efficiency of *SPT*-siRNA with > 60% reduction in *SPT* gene transcripts in the retinal microvessels from normal mice receiving intravitreal injection of *SPT*-siRNA.

#### Rac1–Nox2–ROS signaling

**Figure 6:**
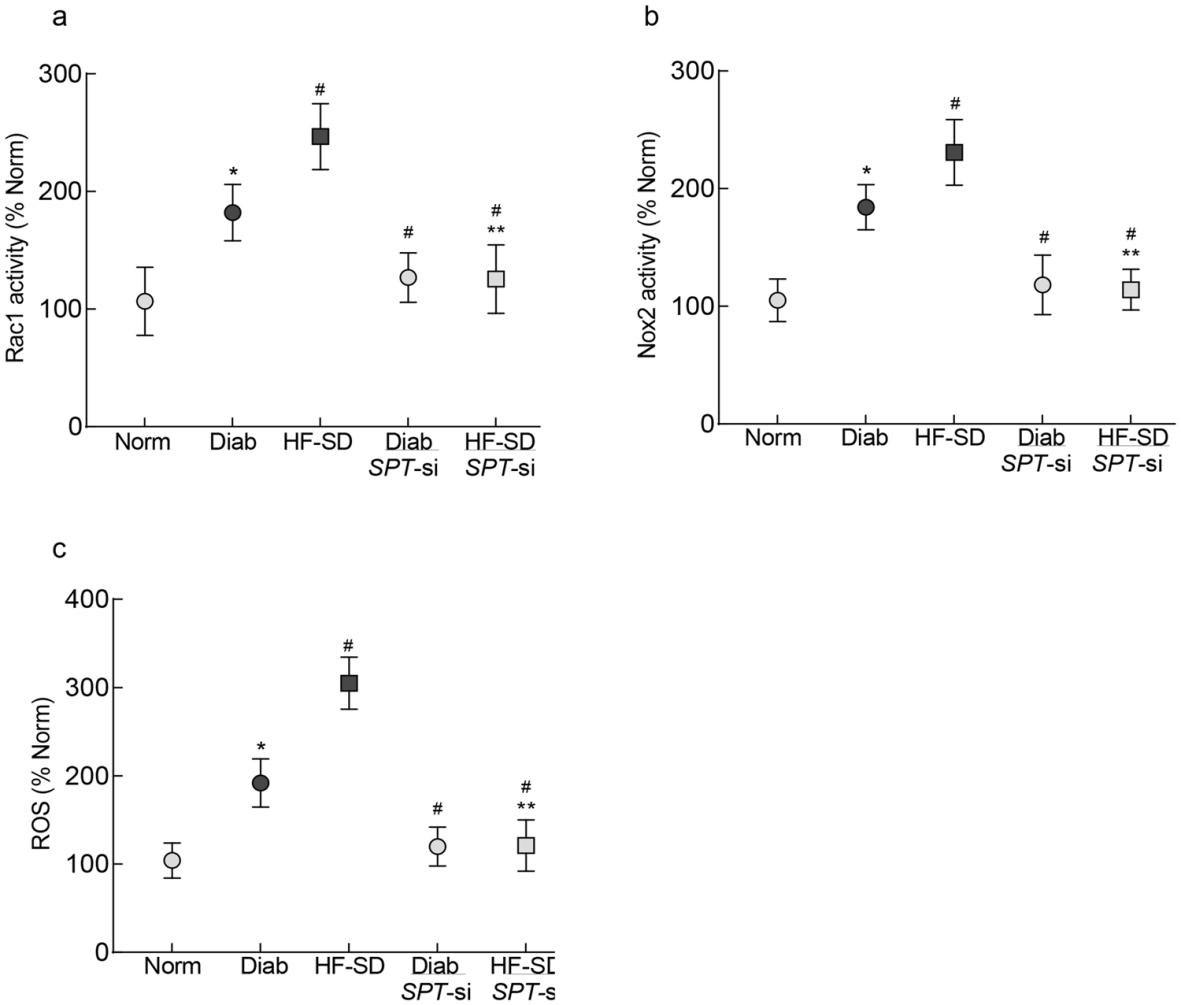
Rac1–Nox2–ROS signaling and SPT inhibition in retinal microvessels from diabetic mice. Retinal microvessels were analyzed for (**a**) Rac1 activity, (**b**) Nox2 activity, and (**c**) ROS by DCHFDA method. Each measurement was made in duplicate, and the values are means ± SD of 5–7 mice/group. Norm = normal mice on regular chow; Diab = diabetic mice on regular chow; HF-SD = diabetic mice on high fat chow; Diab*/SPT*-si or HF-SD/*SPT*-si = *SPT*-siRNA administered diabetic mice on regular chow or high fat chow, respectively. *p < 0.05 vs Norm, ^#^p < 0.05 vs Diab and ^#^**p < 0.05 vs HF-SD.

Consistent with the in vitro results, Rac1–Nox2–ROS axis was activated in the retinal microvessels from mice in Diab group, and Rac1–Nox2 activation and elevation in ROS levels were further increased in HF-SD group (p < 0.05 vs Diab; Fig. 6a–c). Administration of *SPT*-siRNA at the time of induction of diabetes in Diab and HF-SD groups (Diab/*SPT*-si and HF-SD/*SPT*-si groups) prevented activation of Rac1–Nox2 and increase in ROS levels. The values obtained from Diab/*SPT*-si or HF-SD/*SPT*-si groups were significantly different from those in Diab or HF-SD groups (p < 0.05). However, values in Diab/*SPT*-si and HF-SD/*SPT*si were not different from each other.

#### Transcriptional activation of *Rac1*

**Figure 7:**
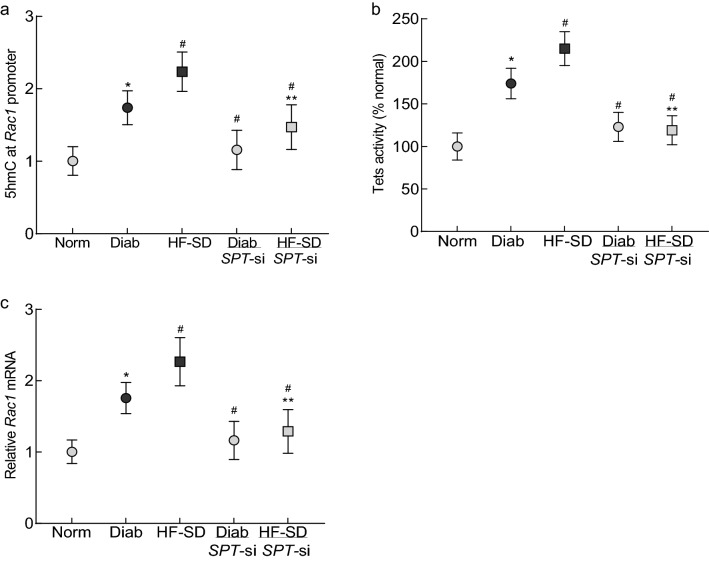
Regulation of SPT and *Rac1* transcriptional activation in diabetic mice. (**a**) 5hmC levels at *Rac1* promoter, (**b**) activity of Tets and (**c**) relative *Rac1* mRNA were measured in retinal microvessels from 5–6 mice/group, and the values are represented as means ± SD. Norm and Diab = normal or diabetic mice on regular chow; HF-SD = diabetic mice on high fat chow; Diab/*SPT*-si or HF-SD/*SPT*-si = diabetic mice on regular chow or high fat chow, respectively, receiving *SPT*-siRNA intravitreally. *p < 0.05 vs Norm, ^#^p < 0.05 vs Diab and ^#^**p < 0.05 vs HF-SD.

Mice in HF-SD group had higher 5hmC at *Rac1* promoter compared to Diab group, and administration of *SPT*-siRNA ameliorated increase in 5hmC; 5hmC values in HF-SD/*SPT*-si group were significantly reduced, compared to HF-SD group (p < 0.05) (Fig. 7a). Although 5hmC in HF-SD/*SPT*-si group was slightly higher than in Diab/*SPT*-siRNA, the values among these two groups did not achieve any statistical significance (p > 0.05). Consistent with 5hmC, *SPT*-siRNA also prevented activation of Tets and increase in *Rac1* gene transcription in both Diab/*SPT*-si and HF-SD/*SPT*-si groups (Fig. 7b,c), and these values were not different from those obtained from Norm group (*p > 0.05).

## Discussion

Oxidative stress plays a significant role in the development of diabetic retinopathy, and increase in cytosolic ROS, produced by the activation of Rac1–Nox2, is an early event, which damages the mitochondria, accelerating loss of capillary cells. Addition of hyperlipidemia in a hyperglycemic milieu, which mimics type 2 diabetes, further exacerbates Rac1–Nox2–ROS activation, and as the duration of diabetes increases, mitochondrial damage worsens, ultimately leading to the capillary cell loss and the development of diabetic retinopathy^8,12^. Hyperglycemia is the main instigator of the development of diabetic retinopathy, but abnormalities in lipid metabolism are also considered as potential risk factors for its development, and lipid-lowering therapy has helped in reducing the number of laser treatments in patients with diabetes with proliferative retinopathy^22–24^. Ceramides are a group of complex bioactive lipids, and are shown to play a central role in cell membrane integrity and cellular stress response, and their metabolism is dysregulated in the animal models of diabetic retinopathy, in particular, the levels of proinflammatory and proapoptotic short-chain ceramides are elevated^16,25^. Here, we show that inhibition of the rate-limiting enzyme of de novo ceramide biosynthesis using *SPT*-siRNA, or a chemical inhibitor, prevents activation of Rac1–Nox2–ROS signaling, mitochondrial damage and cell apoptosis in retinal endothelial cells incubated in high glucose medium, with or without lipids. Regulation of SPT, in addition to preventing functional activation of Rac1, also ameliorates the increase in its gene transcription by modulating dynamic DNA methylation–hydroxymethylation at its promoter, and allowing increased accumulation of 5hmC levels and active transcription. Similar protection of Rac1–Nox2–ROS and *Rac1* promoter dynamic DNA methylation–hydroxymethylation by *SPT*-siRNA is confirmed in streptozotocin-induced diabetic mice on regular (type 1 diabetic model) or high fat diet (type 2 diabetic model). These results suggest that inhibition of SPT in a hyperglycemic milieu, via DNA hydroxymethylation of *Rac1* promoter, augments its transcription and activates Rac1–Nox2–ROS signaling. Increase in ROS damages the mitochondria, and ultimately resulting in the development of diabetic retinopathy.

Diabetic patients generally present abnormalities in their lipid metabolism, and type 2 diabetic patients have higher triglyceride levels compared to type I diabetic patients^26,27^; similar phenomenon is also observed in animal models^7,10^. We have shown that the activation of Rac1–Nox2–ROS signaling is accelerated and exacerbated in the retinal vasculature in type 2 diabetic experimental models compared to type 1 diabetic models. Moreover, simultaneous presence of both hyperlipidemia and hyperglycemia further worsens capillary cell apoptosis and the development of diabetic retinopathy^7,10,17^. Ceramides tend to accumulate in individuals with obesity or dyslipidemia^13^, and are considered as ‘*lipotoxic inducers*’ of metabolic disorders*;* inhibition of their biosynthesis is shown to improve defects in islets in animal models of diabetes^28^. Here, our results show that both *SPT*-siRNA or Myr prevent activation of Rac1 in retinal endothelial cells exposed to high glucose and palmitic acid. Furthermore, activation of Nox2, co-localization of Rac1–Nox2 and ROS levels are also significantly decreased in *SPT*-siRNA transfected cells, suggesting that ceramides play major roles in activation of Rac1–Nox2 signaling. In support, inhibition of biosynthesis of ceramides is shown to improve palmitic acid-induced Nox2-superoxide generation via preventing sustained activation of Rac1 signaling pathway in pancreatic β-cells^29^.

In the pathogenesis of diabetic retinopathy production of ROS by Rac1–Nox2 activation is an early event, and this ROS production in the cytosol precedes mitochondrial damage, further exacerbating accumulation of free radicals. Damaged mitochondrial membranes leak cytochrome *c* into the cytoplasm, activating the apoptotic machinery, and accelerated capillary cell loss is followed by the histopathology characteristic of diabetic retinopathy. Furthermore, sustained increase in ROS damages mtDNA, compromising the electron chain system, which continues to self-propagate the vicious cycle of free radicals^4,8^. Animal models have documented that hyperlipidemia, in a hyperglycemic environment, also accelerates and exacerbates mtDNA damage and cell apoptosis in the retinal vasculature^7,10^. Here, our results show that inhibition of SPT, important in ceramide de novo biosynthesis, prevents mitochondrial damage, as documented by amelioration of decrease in mtDNA transcription of *ND6* of complex I and *Cytb* of complex III, and capillary cell death. Consistent with our results, ceramides are intimately associated with regulation of mitochondrial outer membrane permeability, and increase in their levels, by altering outer mitochondrial membrane permeability, are implicated in the apoptosis^30^. Ceramides are also shown to directly suppress respiratory chain complex I and complex III, increasing ROS production-apoptosis^31–33^, and hypoxia/reoxygenation-induced increased ROS in endothelial cells is linked with ceramide-induced suppression of the mitochondrial respiratory chain^34^*.*

Results presented here show that regulation of ceramides ameliorates increase in *Rac1* transcription, induced by glucotoxic or glucolipotoxic environment. Our previous work has shown that *Rac1* promoter undergoes dynamic DNA methylation; glucotoxic environment increases Dnmt1 binding at *Rac1* promoter, and while 5mC levels become subnormal, 5hmC levels are increased, suggesting a concomitant activation of the hydroxymethylation machinery, which rapidly converts 5mC to 5hmC. Formation of 5hmC facilitates the binding of the transcription factor, and activates *Rac1* transcription. This dynamic DNA methylation–hydroxymethylation is exacerbated in glucolipotoxic environment, further increasing *Rac1* transcription^7,11,12,35^. Our data show that inhibition of SPT also ameliorates increase in hydroxymethylation of *Rac1* promoter and its transcription, and prevents activation of Tets. Although how ceramides activate DNA methylation–hydroxymethylation machinery is not clear, the possible mechanism could be that elevated ceramides levels in diabetes, via increasing ROS, activate DNA methylation–hydroxymethylation machinery, and increase *Rac1* transcription. In support, ROS are shown to also function as catalysts of DNA methylation^36,37^, and ceramide accumulation is associated with increased oxidative stress^38^ and alterations in the DNA methylation status of anti-proliferative genes^39^.

As mentioned above, Rac1–Nox2–ROS activation is an early event in the pathogenesis of diabetic retinopathy, and is seen before mitochondrial damage or capillary cell loss^8,11^. Similar effect of inhibition of SPT on Rac1–Nox2–ROS signaling, and on *Rac1* transcriptional activation, obtained in retinal microvasculature from type 1 (Diab) and type 2 (HF-SD) diabetic animal models, the two models that develop vascular histopathology characteristic of diabetic retinopathy^7,21^, further strengthen the role of ceramides in ROS generation in the early stages of diabetic retinopathy. We acknowledge that our study has focused on ceramide-mediated transcriptional activation of *Rac1*, however, guanine exchange factors Tiam1 and Vav2 are also intimately associated with the functional activation of retinal Rac1 signaling in diabetic retinopathy, and inhibition of guanine exchange factors prevents activation of Rac1–Nox2–ROS signaling and inhibits mitochondrial damage and the development of retinopathy in diabetic mice^40^. Cross-talk between ceramides and Rac1 signaling via regulatory factors, including Tiam1 and Vav2, in the development of diabetic retinopathy, however, cannot be ruled out. Furthermore, our study is focused on the regulation of ceramide de novo biosynthesis enzyme SPT; contributions of other products of SPT pathway, besides ceramide, and the role of other ceramide generation pathways including sphingomyelinase and salvage pathways^41^, in the activation of Rac1–Nox2 signaling in diabetic retinopathy cannot be ruled out.

Thus, in conclusion, we have identified a mechanism of elevated ceramide-mediated further worsening of the development of retinopathy in obese patients with diabetes. Regulation of DNA methylation–hydroxymethylation machinery, by ameliorating ceramide production prevents increased transcription of *Rac1,* regulates the activation of Rac1–Nox2 signaling, protecting the mitochondria from damaging cytosolic ROS, and preventing accelerated capillary cell loss. Thus, for diabetic patients with dyslipidemia, regulation of ceramide levels may be equally important as maintaining their glycemic control, to inhibit/prevent the development of diabetic retinopathy.

## Methods

### Retinal endothelial cells

Human retinal vascular endothelial cells (Cat. no. ACBRI 181, Cell Systems Corp, Kirkland, WA, USA) were cultured in Dulbecco’s modified Eagle medium (DMEM, Cat. no. D5523; Sigma-Aldrich Corp., St. Louis, MO, USA) -F12 supplemented with 15 µg/ml endothelial cell growth supplement, 12% heat-inactivated fetal bovine serum, and 1% of each insulin, transferrin, selenium, GlutaMAX and antibiotic/antimycotic. Cells were grown in a humidified environment of 95% O_2_ and 5% CO_2_ at 37 °C^42^. Confluent cells from 7th to 8th passage were incubated in 5 mM or 20 mM d-glucose for 96 h in the presence or absence of 50 µM palmitic acid (Cat no. P9767; Sigma-Aldrich Corp; HG + PA group)^17^. A group of cells were incubated in high glucose with, or without palmitate, in the presence of an inhibitor of ceramide de novo biosynthesis, Myriocin (5 μM; Cat no. M1177, Sigma-Aldrich)^43^.

Cells from 6th to 7th passage were transfected with *SPT*-siRNA (10 nM, Cat. No. SPTLC1 s20712, Thermo-Fisher, Waltham, MA, USA), employing lipofectamine RNAiMAX (Cat. no. 13778150, Invitrogen, Life Technologies, Carlsbad, CA) in serum free Opti-MEM, and the transfected cells were then incubated in 5 mM or 20 mM d-glucose for 96 h in the absence or presence of palmitate. Parallel incubations with non-targeting scrambled RNA (SC), were used as their controls^44^. To rule out the effect of osmolarity, each experiment included cells incubated in 20 mM l-glucose instead of 20 mM d-glucose. Each experiment was performed in duplicate in three or more different HREC preparations.

### Mice

C57BL/6 mice (body weight, ~ 20 g, both male and female, Jackson Laboratory, Bar Harbor, ME, USA) were divided into two groups; mice in group 1 remained on the regular chow (Cat. no. 7001; Envigo, Indianapolis, IN, USA) containing 4.25% kcal as fat, and mice in group 2 were placed on a high-fat diet (Cat. no. D12451; Research Diets Inc., New Brunswick, NJ, USA) containing 45% kcal as fat. After 8 weeks, mice on high-fat diet were injected a low dose of streptozotocin (30 mg/kg BW i.p.) to induce diabetes (HF-SD group). This diabetic animal model presents hallmark features of type 2 diabetes including insulin resistance, impaired glucose tolerance and hyperlipidemia^7,45^. Simultaneously, a group of mice on regular chow were also injected with streptozotocin (60 mg/kg BW) for four consecutive days (Diab group), and the other half remained as normal controls (Norm group). Mice in HF-SD group were maintained on the same high-fat diet and mice in Diab and Norm groups remained on regular chow throughout the experiment.

For *SPT*-siRNA experiments, soon after induction of diabetes (3 days after the last injection of streptozotocin), a group of mice in Diab and HF-SD groups were anesthetized with ketamine–xylazine (100 mg/kg ketamine and 12 mg/kg xylazine) and using a 32-gauge needle attached to a 5 μl glass Hamilton syringe, 2 μg *SPT*-siRNA (Cat. no. SPTLC1 MSS2 18753, Thermo Fisher), mixed with 2 μl Invivofectamine (Cat. no. IVF 3001, Invitrogen) was administered intravitreally in the left eye under a dissecting microscope (Diab/*SPT*-si and HF-SD/*SPT*-si groups, respectively), as described previously^35^. Mice were sacrificed five weeks after establishment of diabetes, and each of the five groups (Norm, Diab, HF-SD, Diab/*SPT*-si, HF-SD/*SPT*-si) had eight or more mice with similar numbers of males and females. For transfection efficiency, *SPT* expression was quantified by real time quantitative RTPCR (qRT-PCR). The treatment of animals was in accordance with the guidelines of the Association for Research in Vision and Ophthalmology Resolution on the Use of Animals in Research, and the experimental protocols were approved by Wayne State University’s Animal Care and Use Committee. This study is reported in accordance with the guidelines set by ‘Animal research: reporting of in vivo experiments.

Two to three days before termination of the experiment, mice were fasted overnight, and administered glucose (2 g/kg BW, i.p.). Glucose tolerance was determined by measuring their blood glucose (by glucose-oxidase reagent strips), before, and up to 120 min after, glucose administration^7,10^. The severity of hyperglycemia, and the patterns of glucose tolerance tests were similar in both male and female mice in each group.

### Retinal microvessels

Retina was incubated in 4–5 ml of distilled water for 1 h at 37 °C in a shaker water bath. The retinal vasculature was isolated under a microscope by repetitive inspiration and ejection through a Pasteur pipette, and microvessels were washed with PBS^35^.

### Enzyme activities

GTPase activity of Rac1 was determined in 20–25 μg protein using G-LISA colorimetric assay kit (Cat. no. BK-128; Cytoskeleton, Denver, CO, USA), as described previously^40^. Values obtained from HRECs in NG or retinal microvessels from normal mice, were considered as 100%.

Nox2 activity was estimated in 10 μg protein by a chemiluminescence method using 25 μM lucigenin (Cat. no. M8010, Sigma-Aldrich) as an electron acceptor and 200 μM NADPH as a substrate^40^. Normal glucose/mice values were considered as 100%.

Activity of Tets was quantified in 20 μg nuclear protein by employing a 5mC-Hydroxylase TET Activity/Inhibition Assay Kit (Cat. no. P-3087, EPIGENTEK, Farmingdale, NY, USA), as reported previously^46^. Values from cells in normal glucose or retinal microvessels from normal mice were considered as 100%.

### Reactive oxygen species

Protein (5 μg) was incubated with 4 μM DCHFDA (2′,7′-dichlorofluorescein diacetate; Cat. no. D6883; Sigma-Aldrich) in dark for 20 min, and the resultant fluorescence was measured at 485 nm and 530 nm as excitation and emission wavelengths, respectively^35^. The values are represented as percentage, considering normal glucose or normal mice values as 100%.

### Immunofluorescence

Rac1 and Nox-2 colocalization was performed by immunofluorescence technique using primary antibodies against Rac1 (Cat. no. PA1-091x, Invitrogen, 1:200 dilution), and Nox-2 (Cat. no. Ab-43801, Abcam, Cambridge, MA, USA, 1:200 dilution), and their secondary antibodies were DyLight green- or Texas red-conjugated respectively (1:500 dilution each). 4′,6-diamidino-2-phenylindole (DAPI) containing mounting media (blue; Cat. no. H-1000 Vector Laboratories, Burlingame, CA, USA) was used to mount the coverslips, and the images were taken under a ZEISS ApoTome fluorescence microscope (Carl Zeiss, Chicago, IL, USA) using 20 × objective^17^. The ‘Arithmetic mean intensity’ (AMI) of Rac1 and Nox2, and Pearson correlation coefficient between them were quantified using Zeiss software module^47^.

### Gene transcripts

Total RNA was extracted with TRIZOL reagent (Invitrogen) and cDNA was prepared from 1 μg RNA using the High-Capacity cDNA Reverse Transcription Kit (Applied Biosystems, Foster City, CA, USA). Products specificity was confirmed by SYBR green single melt curve analysis. β-Actin (human) or 18S (mice) were used as housekeeping genes, and each sample was analyzed in triplicate by qRT-PCR. The relative fold change was calculated using delta delta Ct method^12,17^. The primer sequences are listed in Table 1.

**Table 1:** Primer sequences.

Primer	Sequence
**Human**	
*Rac1*	Fwd—CGCCCCCTATCCTATCCGCA
	Rev—GAACACATCGGCAATCGGCTTGT
*SPT*	Fwd—AAGTATGGCGTGGGGACTTG
	Rev—GTTGGCCTGTTCCCGGATTA
*ND6*	Fwd—CCAAGACCTCAACCCCTGAC
	Rev—GGTGTGGTCGGGTGTGTTAT
*Cytb*	Fwd—TCACCAGACGCCTCAACCGC
	Rev—GCCTCGCCCGATGTGTAGGA
*Rac1* promoter (− 113 to 30)	Fwd—CTTCCGAGCATTCCCGAAGTC
	Rev—AATGGCCGCTCCACTCAC
*β-Actin*	Fwd—AGCCTCGCCTTTGCCGATCCG
	Rev—TCTCTTGCTCTGGGCCTCGTCG
**Mice**	
*Rac1*	Fwd—CACGACCAATGCATTTCCTGG
	Rev—AAGAACACGTCTGTCTGCGG
*SPT*	Fwd—CAGGAGCGTTCTGATCTTACAG
	Rev—CCGGACACGATGTTGTAGTT
*Rac1* promoter (− 957 to − 1061)	Fwd—CGGAACCCCGTGGTCAATAA
	Rev—CCCACAAGACGACAGGGAAA
*18S*	Fwd—GCCCTGTAATTGGAATGAGTCCACTT
	Rev—CTCCCCAAGATCCAACTACGAGCTTT

### Cell death

Cell death was quantified using Cell Death Detection ELISA PLUS kit from Roche Diagnostics (Cat. No. 11774425001; Indianapolis, IN, USA), as described previously^36,37^. The results were calculated as percentage considering the values obtained from cells in NG as 100%.

### Hydroxymethyl cytosine

Using primers for *Rac1* promoter and Hydroxymethylated DNA Immunoprecipitation kit (Cat. no. P-1038, EPIGENETEK), 5hmC levels were quantified in the total genomic DNA isolated from HRECs or retinal microvessels^7^. The values are represented as fold change considering normal glucose or normal mice values as 1.

### Dnmt1 binding at *Rac1* promoter

Chromatin immunoprecipitation (ChIP) technique was employed to assess Dnmt1 binding. Cells fixed for 10 min with 1% paraformaldehyde, followed by chromatin extraction, were sonicated in ChIP lysis buffer to generate 200–500 bp fragments. Protein-DNA complex (100 μg) was immunoprecipitated overnight at 4 °C with Dnmt1 antibody; normal rabbit IgG was used as an antibody control (Cat. no. Ab 13537 and Cat. no. Ab 171870 respectively, Abcam). Protein A/G agarose beads (EMD Millipore, Billerica, MA) were employed to pull down the antibody-chromatin complexes. Protein-associated DNA was recovered by incubating the eluent at 65 °C, purified by phenol extraction, and precipitated with ethanol. Dnmt1 binding was quantified by qRT-PCR using primers specific for *Rac1* promoter region^12^. The values obtained from cells in normal glucose, or retinal microvessels from normal mice, are considered as 1.

### Statistical analysis

The results are expressed as mean ± SD, and employing Graph Pad Prism 9.3.1 (San Diego, CA), significance of variance was analyzed by ‘one-way ANOVA’, followed by ‘Dunn post hoc’ test. p value < 0.05 was considered as statistically significant.

## Data Availability

All data generated or analyzed during this study are included in this article, and RAK is the guarantor of this work and takes responsibility for the integrity of the data and the accuracy of the data analysis.

## References

[1] Aiello LM (2003). Perspectives on diabetic retinopathy. Am. J. Ophthalmol..

[2] Frank RN (2004). Diabetic retinopathy. N. Engl. J. Med..

[3] Diabetes Control and Complications Trial Research Group (1993). The effect of intensive treatment of diabetes on the development of long-term complications in insulin-dependent diabetes mellitus. N. Engl. J. Med..

[4] Kowluru RA, Kowluru A, Mishra M, Kumar B (2015). Oxidative stress and epigenetic modifications in the pathogenesis of diabetic retinopathy. Prog. Retin. Eye Res..

[5] Kowluru RA, Mishra M (2015). Oxidative stress, mitochondrial damage and diabetic retinopathy. Biochem. Biophys. Acta..

[6] Frank RN (2015). Diabetic retinopathy and systemic factors. Middle East Afr. J. Ophthalmol..

[7] Kowluru RA (2020). Retinopathy in a diet-induced type 2 diabetic rat model, and role of epigenetic modifications. Diabetes.

[8] Kowluru RA (2021). Diabetic retinopathy and NADPH oxidase-2: A sweet slippery road. Antioxidants (Basel).

[9] Kowluru RA, Kowluru A, Veluthakal R, Mohammad G, Syed I, Santos JM, Mishra M (2014). TIAM1-RAC1 signalling axis-mediated activation of NADPH oxidase-2 initiates mitochondrial damage in the development of diabetic retinopathy. Diabetologia.

[10] Kowluru RA, Mishra M, Kowluru A, Kumar B (2016). Hyperlipidemia and the development of diabetic retinopathy: Comparison between type 1 and type 2 animal models. Metabolism.

[11] Kowluru RA, Mishra M, Kumar B (2016). Diabetic retinopathy and transcriptional regulation of a small molecular weight G-Protein, Rac1. Exp. Eye Res..

[12] Kowluru RA, Radhakrishnan R, Mohammad G (2021). Regulation of Rac1 transcription by histone and DNA methylation in diabetic retinopathy. Sci. Rep..

[13] Summers SA, Chaurasia B, Holland WL (2019). Metabolic messengers: Ceramides. Nat. Metab..

[14] Choi RH, Tatum SM, Symons JD, Summers SA, Holland WL (2021). Ceramides and other sphingolipids as drivers of cardiovascular disease. Nat. Rev. Cardiol..

[15] Levitsky Y, Hammer SS, Fisher KP, Huang C, Gentles TL, Pegouske DJ, Xi C, Lydic TA, Busik JV, Proshlyakov DA (2020). Mitochondrial ceramide effects on the retinal pigment epithelium in diabetes. Int. J. Mol. Sci..

[16] Busik JV (2021). Lipid metabolism dysregulation in diabetic retinopathy. J. Lipid Res..

[17] Kumar B, Kowluru A, Kowluru RA (2015). Lipotoxicity augments glucotoxicity-induced mitochondrial damage in the development of diabetic retinopathy. Investig. Ophthalmol. Vis. Sci..

[18] Hannun YA, Obeid LM (2008). Principles of bioactive lipid signalling: Lessons from sphingolipids. Nat. Rev. Mol. Cell Biol..

[19] Chaurasia B, Summers SA (2021). Ceramides in metabolism: Key lipotoxic players. Annu. Rev. Physiol..

[20] Sasset L, Di Lorenzo A (2022). Sphingolipid metabolism and signaling in endothelial cell functions. Adv. Exp. Med. Biol..

[21] Mizutani M, Kern TS, Lorenzi M (1996). Accelerated death of retinal microvascular cells in human and experimental diabetic retinopathy. J. Clin. Investig..

[22] Chew EY, Klein ML, Ferris FL, Remaley NA, Murphy RP, Chantry K, Hoogwerf BJ, Miller D (1996). Association of elevated serum lipid levels with retinal hard exudate in diabetic retinopathy. Early Treatment Diabetic Retinopathy Study (ETDRS) Report 22. Arch. Ophthalmol..

[23] Keech AC, Mitchell P, Summanen PA, O'Day J, Davis TM, Moffitt MS, Taskinen MR, Simes RJ, Tse D, Williamson E (2007). Effect of fenofibrate on the need for laser treatment for diabetic retinopathy (FIELD study): A randomised controlled trial. Lancet.

[24] Amutha A, Pradeepa R, Chella KS, Anjana RM, Unnikrishnan R, Mohan V (2017). Lipid profile in childhood-and youth-onset type 2 diabetes and their association with microvascular complications. J. Assoc. Physicians India.

[25] Chakravarthy H, Navitskaya S, O'Reilly S, Gallimore J, Mize H, Beli E, Wang Q, Kady N, Huang C, Blanchard GJ (2016). Role of acid sphingomyelinase in shifting the balance between proinflammatory and reparative bone marrow cells in diabetic retinopathy. Stem Cells.

[26] Khiari M, Zribi S, Zahra H, Faten M, Olfa B, Jamoussi H (2018). Dyslipidemia: Type 1 diabetes vs type 2 diabetes. Endocr. Rev..

[27] Hirano T (2018). Pathophysiology of diabetic dyslipidemia. J. Atheroscler. Thromb..

[28] Shimabukuro M, Zhou YT, Levi M, Unger RH (1998). Fatty acid-induced beta cell apoptosis: A link between obesity and diabetes. Proc. Natl. Acad. Sci. U.S.A..

[29] Syed I, Jayaram B, Subasinghe W, Kowluru A (2010). Tiam1/Rac1 signaling pathway mediates palmitate-induced, ceramide-sensitive generation of superoxides and lipid peroxides and the loss of mitochondrial membrane potential in pancreatic beta-cells. Biochem. Pharmacol..

[30] Ueda N (2015). Ceramide-induced apoptosis in renal tubular cells: A role of mitochondria and sphingosine-1-phoshate. Int. J. Mol. Sci..

[31] Gudz TI, Tserng KY, Hoppel CL (1997). Direct inhibition of mitochondrial respiratory chain complex III by cell-permeable ceramide. J. Biol. Chem..

[32] Andrieu-Abadie N, Gouazé V, Salvayre R, Levade T (2001). Ceramide in apoptosis signaling: Relationship with oxidative stress. Free Radic. Biol. Med..

[33] Yu J, Novgorodov SA, Chudakova D, Zhu H, Bielawska A, Bielawski J, Obeid LM, Kindy MS, Gudz TI (2007). JNK3 signaling pathway activates ceramide synthase leading to mitochondrial dysfunction. J. Biol. Chem..

[34] Therade-Matharan S, Laemmel E, Carpentier S, Obata Y, Levade T, Duranteau J, Vicaut E (2005). Reactive oxygen species production by mitochondria in endothelial cells exposed to reoxygenation after hypoxia and glucose depletion is mediated by ceramide. Am. J. Physiol. Regul. Integr. Comp. Physiol..

[35] Duraisamy AJ, Mishra M, Kowluru A, Kowluru RA (2018). Epigenetics and regulation of oxidative stress in diabetic retinopathy. Investig. Ophthalmol. Vis. Sci..

[36] Wu Q, Ni X (2015). ROS-mediated DNA methylation pattern alterations in carcinogenesis. Curr. Drug Targets.

[37] Scarpato R, Testi S, Colosimo V, Garcia Crespo C, Micheli C, Azzarà A, Tozzi MG, Ghirri P (2020). Role of oxidative stress, genome damage and DNA methylation as determinants of pathological conditions in the newborn: An overview from conception to early neonatal stage. Mutat. Res. Rev. Mutat. Res..

[38] Bekhite M, González-Delgado A, Hübner S, Haxhikadrija P, Kretzschmar T, Müller T, Wu JMF, Bekfani T, Franz M, Wartenberg M (2021). The role of ceramide accumulation in human induced pluripotent stem cell-derived cardiomyocytes on mitochondrial oxidative stress and mitophagy. Free Radic. Biol. Med..

[39] Castro K, Ntranos A, Amatruda M, Petracca M, Kosa P, Chen EY, Morstein J, Trauner D, Watson CT, Kiebish MA (2019). Body mass index in multiple sclerosis modulates ceramide-induced DNA methylation and disease course. EBioMedicine.

[40] Mohammad G, Duraisamy AJ, Kowluru A, Kowluru RA (2019). Functional regulation of an oxidative stress mediator, Rac1, in diabetic retinopathy. Mol. Neurobiol..

[41] Skácel J, Slusher BS, Tsukamoto T (2021). Small molecule inhibitors targeting biosynthesis of ceramide, the central hub of the sphingolipid network. J. Med. Chem..

[42] Mishra M, Duraisamy AJ, Bhattacharjee S, Kowluru RA (2019). Adaptor protein p66Shc: A link between cytosolic and mitochondrial dysfunction in the development of diabetic retinopathy. Antioxid. Redox Signal..

[43] Suzuki J, Akahane K, Nakamura J, Naruse K, Kamiya H, Himeno T, Nakamura N, Shibata T, Kondo M, Nagasaki H (2011). Palmitate induces apoptosis in Schwann cells via both ceramide-dependent and independent pathways. Neuroscience.

[44] Mohammad G, Kowluru RA (2021). Nuclear genome-encoded long noncoding RNAs and mitochondrial damage in diabetic retinopathy. Cells.

[45] Reed MJ, Meszaros K, Entes LJ, Claypool MD, Pinkett JG, Gadbois TM, Reaven GM (2000). A new rat model of type 2 diabetes: The fat-fed, streptozotocin-treated rat. Metabolism.

[46] Kowluru RA, Shan Y, Mishra M (2016). Dynamic DNA methylation of matrix metalloproteinase-9 in the development of diabetic retinopathy. Lab. Investig..

[47] Adler J, Parmryd I (2010). Quantifying colocalization by correlation: The Pearson correlation coefficient is superior to the Mander's overlap coefficient. Cytom. A.

